# How Much Can the Genotype Predict Phenotypical Power Performance in Elite Male and Female Athletes?

**DOI:** 10.5114/jhk/190679

**Published:** 2024-12-06

**Authors:** Lukas Reichert, Sebastian Hacker, Michael Mutz, Markus Raab, Lena Wiese, Karsten Krüger, Karen Zentgraf

**Affiliations:** 1 Movement and Exercise Science, Goethe University Frankfurt, Frankfurt, Germany.; 2 Department of Exercise Physiology and Sports Therapy, Justus Liebig University Giessen, Giessen, Germany.; 3 Social Sciences of Sport, Justus Liebig University Giessen, Giessen, Germany.; 4 Institute of Psychology, German Sport University Cologne, Cologne, Germany.; 5 Database Technologies and Data Analytics, Goethe University Frankfurt, Frankfurt, Germany.

**Keywords:** expertise, sport genetics, elite sport

## Abstract

The role of power performance in elite athletes has been enriched by identifying associations between specific single nucleotide polymorphisms (SNPs) and power performance. To deepen our understanding of this association, the objective of this study was to explore the relationship between the genotype and the phenotype in elite athletes. A total of 278 German national squad athletes (156 males, 122 females) underwent genotyping, and their performance in a countermovement jump test (CMJ) and 10-m sprint was assessed. Genotype distribution was analyzed using Chi-square tests. Spearman correlation was employed to examine associations between selected SNPs (e.g., ACTN3, AGT, HSD17B14, IP6K3, MTRR, UCP2, and VDR) and CMJ and sprint performances. Gender-specific polygenic “Total Genotype Scores” (TGSsig) were calculated. Predictive power of TGSsig on power performance was evaluated using linear regression. TGSsig explained 10% of variance in CMJ and sprint performance in both genders. Among males, correlations were identified between AGT and VDR with the CMJ as well as between IP6K3 and sprint performance (p < 0.05). In females, ACTN3, AGT, and UCP2 exhibited associations with the CMJ, while HSD17B14, MTRR, and UCP2 were correlated with sprint performance (p < 0.05). Significant differences in genotype distribution between genders were observed for DMD and MPRIP. Our findings strengthen the idea of power being partly heritable, however, the genotype only partially, by 10%, determines power performance. The role of the athletes' genotype for individual performance development should be investigated in future longitudinal studies.

## Introduction

While early studies in expertise research focused on the idea of deliberate practice directly leading to expertise (Ericsson et al., 1993), newer models focus more on multifactorial approaches. In the *Multifactorial Gene-Environment Interaction Model of Expertise*, Ullén et al. (2016) consider several factors such as cognition and physical properties as well as the gene-environment interaction as important. This framework has been recently applied to expertise in sports (Zentgraf and Raab, 2023) as part of a government-funded project (the in:prove project) which aims to develop performance and health on an individual basis. Based on this framework, the current paper aims to investigate the relationship of selected genetic polymorphisms and phenotypical power performance in an elite athletes’ population.

Power is defined as the ability to generate maximum force within the shortest possible time, with studies highlighting its importance in team sports and gymnastics (for review see Cronin and Sleivert, 2005). In volleyball, Gonçalves et al. (2021) demonstrated that elite players exhibited superior power performance in countermovement jump tests and the medicine ball throw compared to sub-elite players. In ice hockey, Vigh-Larsen et al. (2019) as well as Ransdell et al. (2011) have concluded that elite-level ice hockey requires a high level of power for both male and female athletes. In gymnastics, Douda et al. (2008) showed that power was an important determinant of successful performance. Therefore, power seems to be a relevant performance variable in such sports.

In the field of genetic research, previous studies have suggested that genotypes of specific single nucleotide polymorphisms (SNPs), such as the RR genotype in Actinin-alpha 3 R577X (ACTN3) and the deletion genotype in the angiotensin I converting enzyme insertion/deletion polymorphism (ACE), are over-represented within elite athletes in power-oriented sports (for review see El Ouali et al., 2024; Ma et al., 2013). These SNPs are also significantly associated with phenotypical power performance measures such as the countermovement jump (for review see Ahmetov et al., 2022; Appel et al., 2021; Varillas Delgado et al., 2022). The reported associations indicate that part of the variance in power performance may be explained by the athletes’ genotype and is therefore partly heritable (49–86% according to Ahmetov et al., 2022). One of the best studied gene variants in this context is the R577X polymorphism of the ACTN3 gene (rs1815739; Del Coso et al., 2019). ACTN3 is responsible for encoding the protein alpha-actinin-3, which is primarily found in fast-twitch fibers in the Z-line of skeletal muscles favoring the ability to generate strong and powerful muscle contractions. Depending on the genotypical expression of ACTN3, alpha-actinin 3 is encoded. While a homozygous XX genotype encodes a stop-codon and therefore does not lead to expression of the protein, the homozygous RR genotype leads to expression of alpha-actinin 3. Accordingly, knowledge of the genotype allows conclusions to be drawn, for example, about muscle fiber properties. For instance, in ACTN3, previous studies have shown that male and female elite sprint athletes have significantly higher frequencies of the R allele than controls (Yang et al., 2003).

In their review, Maciejewska-Skrendo et al. (2019) described further SNPs and their physiological background for associations with power performance, of which some are also included in this paper: these are SNPs associated with skeletal muscle structure and function (e.g., the Dystrophin - DMD - rs939787 polymorphism or the Myosin phosphatase Rho interacting protein - MPRIP - rs6502557 polymorphism), involved in blood pressure control (e.g., the ACE or the Angiotensinogen - AGT - Met235Thr polymorphism), that are regulators of energy metabolism and cellular homeostasis (e.g., the Uncoupling protein 2 - UCP2 - Ala55Val or the Hydroxysteroid 17-beta dehydrogenase - HSD17B14 - rs7247312 polymorphism), as well as SNPs encoding factors that control gene expression by rearrangement of chromatin fibers and mRNA stability (e.g., the 5-Methyltetrahydrofolate-homocysteine methyltransferase reductase - MTRR - A66G polymorphism) or by modulating cellular signaling pathways (e.g., Inositol hexakisphosphate kinase 3 - IP6K3 - rs6942022 polymorphism).

In addition to investigating the relationship between individual SNPs, more recent studies use polygenic scores to predict power performance as a complex trait (McAuley et al., 2024). For example, Ruiz et al. (2010a) compared polygenic scores including multiple SNPs such as ACE, ACTN3, and AGT between elite track and field power athletes and non-athletic controls, finding a significantly higher score in power-related athletes. Also, Petr et al. (2022) explained 26% of the variance in jump performance and isokinetic strength using a polygenic score regression. Recent studies indicate that the relationship between the athletes’ genotype and performance phenotypes may differ between genders. Willems et al. (2017), for instance, found stronger associations between a polygenic score and grip strength in males compared to females. To capture potential gender differences in our data, we calculated gender-specific polygenic scores and conducted analyses separately for males and females.

In summary, several studies have investigated the role of SNPs in power performance (Ahmetov et al., 2022). Nonetheless, studies often differ in terms of their methodology (e.g., statistical analysis or polygenic score calculation). Earlier studies focused primarily on frequency-based approaches comparing elite- vs. non-elite athletes in power-related sports without objectifying the phenotype. Also, some studies still provide contradictory findings in the relationship between the genotype and the phenotype (Yang et al., 2023). As argued by Zentgraf and Raab (2023), the genotype is not linearly related to the phenotype, the relationship between both may be altered by epigenetics, environmental factors, training volume/content as well as nutrition (Guest et al., 2019). However, the authors suggest that knowledge of the relationship between the genotype and the phenotype could be used for individualized training prescriptions in elite sports. To enhance the understanding of the interaction between the genotype and the phenotype, this study aimed to answer the question to what extent the genotype can predict phenotypical power performance in elite athletes. Therefore, we used a candidate-gene approach including 23 SNPs that have already been related to power performance in previous studies. A comprehensive overview of the investigated SNPs can be found in Table 1. Based on the literature provided in Table 1, we expected to find correlations between the included SNPs and power performance. Furthermore, based on Petr et al. (2022), we expected to explain some variance in power performance using polygenic scores.

**Table 1:** Single nucleotide polymorphisms associated with power performance including the literature-based genetic score count.

Symbol	Gene	Locus	Polymorphism	Genetic score count (0, 1, 2)	Reference
ACE	Angiotensin I converting enzyme	17q23.3	rs4341 C/G	CC, CG, GG	Puthucheary et al., 2011
ACTN3	Actinin-alpha 3	11q13.1	rs1815739 T/C	TT, CT, CC	Yang et al., 2003
ADRB2	Adrenoceptor beta 2	5q31-q32	rs1042713 A/G	AA, AG, GG	Sawczuk et al., 2013
AGT	Angiotensinogen	1q42.2	rs699 A/T	AA, AG, GG	Zarębska et al., 2013
COTL1	Coactosin-like protein	16q24.1	rs7458 G/A	GG, AG, AA	Maciejewska-Skrendo et al., 2019
CPNE5	Copine V	6p21.2	rs3213537 T/C	TT, TC, CC	Guilherme et al., 2021
DMD	Dystrophin	Xp21.2	rs939787 G/A	GG, AG, AA	Ahmetov and Fedotovskaya, 2015
HIF1A	Hypoxia inducible factor 1 subunit alpha	14q23.2	rs11549465 C/T	CC, TC, TT	Eynon et al., 2010
HSD17B14	Hydroxysteroid 17-beta dehydrogenase 14	19q13.33	rs7247312 A/G	AA, AG, GG	Pickering et al., 2019
IGF1	Insulin-like growth factor 1	12q23.2	rs35767 G/A	GG, AG, AA	Ben-Zaken et al., 2013
IL6	Interleukin 6	7p21	rs1800795 C/G	CC, GC, GG	Ruiz et al., 2010b
IP6K3	Inositol hexakisphosphate kinase 3	6p21.31	rs6942022 T/C	TT, TC, CC	Maciejewska-Skrendo et al., 2019
ITPR1	Inositol 1,4,5-triphosphate receptor type 1	3p26.1	rs1038639 G/T	GG, TG, TT	Moreland et al., 2022
MPRIP	Myosin phosphatase Rho interacting protein	17p11.2	rs6502557 G/A	GG, AG, AA	Maciejewska-Skrendo et al., 2019
MTHFR	Methylenetetrahydrofolate reductase	1p36.22	rs1801131 T/G	TT, TG, GG	Zarębska et al., 2014
MTR	5-Methyltetrahydrofolate-homocysteine methyltransferase	1q43	rs1805087 A/G	AA, AG, GG	Terruzzi et al., 2011
MTRR	5-Methyltetrahydrofolate-homocysteine methyltransferase reductase	5p15.31	rs1801394 A/G	AA, AG, GG	Terruzzi et al., 2011
NOS3	Nitric oxide synthase 3	7q36.1	rs2070744 C/T	CC, CT, TT	Gómez-Gallego et al., 2009a
PPARA	Peroxisome proliferator-activated receptor alpha	22q13.31	rs4253778 G/C	GG, CG, CC	Maciejewska-Skrendo et al., 2021
PPARG	Peroxisome proliferator-activated receptor gamma	3p25.2	rs1801282 C/G	CC, CG, GG	Drozdovska et al., 2013
TRHR	Thyrotropin-releasing hormone receptor	8q23.1	rs7832552 C/T	CC, TC, TT	Miyamoto-Mikami et al., 2017
UCP2	Uncoupling protein 2	11q13.4	rs660339 A/G	AA, AG, GG	Sessa et al., 2011
VDR	Vitamin D receptor	12q13.11	rs1544410 C/T	CC, TC, TT	Bozsodi et al., 2016

Note: The genetic score count is based on the cited reference. Since we found opposite correlations for UCP2 and MTRR, the score for UCP2 and MTRR was inverted before it was used for calculating the polygenic score (see the results section).

## Methods

### Participants

Two hundred seventy-eight (278) professional athletes (age_male_ = 18.72 ± 3.31 years, age_female_ = 18.08 ± 4.12 years; 3 x 3 basketball *n* = 18 male, *n* = 20 female; ice hockey *n* = 65 male, *n* = 23 female; gymnastics *n* = 18 female; trampoline *n* = 13 male, *n* = 12 female; volleyball *n* = 60 male, *n* = 49 female) participated in this study. Athletes were included if they were part of the national squad and were excluded in the event of an injury at the time of testing. Prior to testing, athletes received detailed written and verbal information about the potential benefits and risks associated with this study. Written consent was obtained from each participant (additionally from parents for minors). The study protocol was approved by the Institutional Ethics Committee of the Justus Liebig University Giessen (ethical approval number: AZ 55/22; approval date: 10 May 2022) and was in accordance with the Declaration of Helsinki for human research.

### Study Approach

The present study was conducted using a cross-sectional design to investigate the relationship between genes and power performance. To assess power performance, a 10-m sprint as well as a countermovement jump test (CMJ) were performed. All tests were performed between February 2022 and August 2023. At the beginning of the measurement, blood samples for subsequent DNA analysis were taken. After this, athletes warmed up individually (running, mobility, dynamic stabilization, and coordination tasks) and data in measures were acquired in permuted order as described below.

### Candidate Genes and Polymorphism Selection

**For the present study, a candidate-gene approach was used including *n* = 23 SNPs that already had been associated with power performance in previous studies (Table 1)**.

### Genotyping

DNA was extracted from human whole blood samples using the Chemagic Magnetic Separation Module I (Perkin Elmer Chemagen Technology Inc., Baesweiler, Germany). In a next step, genotyping was performed using the Illumina Global Screening Array + Medical Disease + Psych content (GSAv3.0 + MD + Psych; Illumina Inc, San Diego, CA, USA). All laboratory procedures were conducted according to the manufacturer's instructions. SNP array raw data were then uploaded into, and genotypes were exported from the GenomeStudio2.0 software (Illumina, USA).

### Total Genotype Score Calculation

For polygenic analyses, gender-specific polygenic scores were calculated (based on the work of Williams and Folland, 2008). For this purpose, genotypes were scored from 0 to 2 in relation to their contribution to power performance based on previous studies (Table 1). The homozygous genotype favoring power performance received a score of 2, a score of 1 represented the heterozygous type and a score of 0 related to the homozygous alternative. The SNPs were then summed and transformed into a 0−100 scale by dividing the total score by the maximum possible score and multiplying by 100:


$$TGSsig=\frac{100}{\left(2*n\right)}*\left(GS_{1}+GS_{2}+\ldots +GS_{n}\right)$$

According to previous research (Petr et al., 2022), we calculated polygenic scores only with those SNPs that were significantly correlated with power performance in our own analyses (either with sprint or jump performance), named “Total Genotype Score significant” (TGSsig) which we used for polygenic analyses.

### Power Performance Measures

For power performance assessment, athletes performed a 10-m sprint as well as a CMJ since in both diagnostics maximum force needs to be generated as fast as possible (Markovic et al., 2004; Mero et al., 1992). Athletes performed two test trials for each measurement.

#### Jump Performance

For the evaluation of jump performance, a CMJ was utilized. Athletes’ jump height in cm was assessed using the OptoGait system (Microgate Italy, Bolzano, Italy). Athletes were asked to always keep their hands on their hips. Additionally, they were asked to jump as high as possible after a prior countermovement. Two trials were performed. A third trial was performed if athletes did not perform the previous jump correctly (e.g., the hands were not kept on the hips) or if both trials differed by more than 10% (this was the case in less than 5% of all trials). The trial with the maximum jump height was used for further analysis.

#### Sprint Performance

Linear 10-m sprint times were assessed using Microgate timing gates (Microgate Italy, Bolzano, Italy). Athletes were asked to start in a standardized position (small step, heels on the ground, arms hanging down to the ground) 1 m behind the start line as well as to sprint maximally past the 10-m timing gate. Two trials were performed. If an athlete did not perform the trial correctly (e.g., leaving the standardized position before sprinting) or if both trials differed by more than 10%, a third trial was performed (this was the case in less than 5% of all trials). The rest interval between the subsequent trials equaled one minute. The trial with the best (e.g., shortest) sprint time was used for further analysis.

### Statistical Analysis

All statistical analyses were conducted using SPSS version 26 for Macintosh (IBM Corporation, Armonk, USA). Chi-square tests (χ^2^) were performed to check for Hardy-Weinberg equilibrium as well as to evaluate genotype distributions between genders. For Chi-square tests, Bonferroni correction was used for adjusting *p*-values (with the level of significance set at *p* < 0.002). To examine the relationship between single SNPs and power performance, a Spearman correlation using Spearman’s Rho (ρ) was conducted. To investigate polygenic influence on power performance variables, TGSsig was calculated. The relationship between TGSsig and the CMJ as well as 10-m sprint performance was examined using Pearson correlation analyses (Pearson’s *r)*. A linear regression model was used to explore the predictive role of TGSsig in power performance with the CMJ and sprint performance as dependent variables. Effect sizes were interpreted according to Cohen (1988). The analyses were performed for each power performance variable separately by gender, for both male and female athletes. The level of significance for correlation and regression analyses was set at *p* < 0.05.

## Results

### Genotype Distribution (between Genders)

Frequencies of the studied SNPs are summarized in Table 2. After Bonferroni correction, genotype distribution was in accordance with the Hardy-Weinberg equilibrium (*p* > 0.002). Significant differences in genotype distribution between genders were shown in the frequency in DMD (χ^2^(2) = 72.02, *p* < 0.001, φ = 0.51) with *n* = 0 males and *n* = 40 females exhibiting the heterozygous AG genotype and *n* = 40 males and *n* = 5 females showing the homozygous AA genotype as well as in MPRIP (χ^2^(2) = 13.29, *p* = 0.001, φ = 0.22) with *n* = 52 males and *n* = 20 females exhibiting the heterozygous AG genotype.

**Table 2:** Genotype frequencies of all SNPs between male and female athletes.

Gene variant (SNP)	Genotype	Male (n = 156)	Female (n = 122)	*p*
ACE (*n*/%)	CC, CG, GG	41 (26.3%), 82 (52.6%), 33 (21.2%)	28 (23.0%), 63 (51.6%), 31 (25.4%)	0.652
ACTN3 (*n*/%)	TT, CT, CC	24 (15.4%), 73 (46.8%), 59 (37.8%)	21 (17.2%), 59 (48.4%), 42 (34.4%)	0.821
ADRB2 (*n*/%)	AA, AG, GG	32 (20.5%), 59 (37.8%), 65 (41.7%)	16 (13.1%), 57 (46.7%, 48 (39.3%)	0.169
AGT (*n*/%)	AA, AG, GG	42 (27.6%), 79 (50.6%), 34 (21.8%)	44 (36.1%), 51 (41.8%), 27 (22.1%)	0.256
COTL1 (*n*/%)	GG, AG, AA	122 (78.2%), 32 (20.5%), 2 (1.3%)	96 (78.7%), 24 (19.7%), 2 (1.6%)	0.958
CPNE5 (*n*/%)	TT, TC, CC	4 (2.6%), 39 (25.0%), 113 (72.4%)	3 (2.5%), 24 (19.7%), 95 (77.9%)	0.568
**DMD** (*n*/%)	GG, AG, AA	116 (74.4%), 0 (0%), 40 (25.6%)	77 (63.1%), 40 (32.8%), 5 (4.1%)	**< 0.001**
HIF1A (*n*/%)	CC, TC, TT	131 (84.0%), 24 (15.4%), 1 (0.6%)	97 (79.5%), 22 (18.0%), 3 (2.5%)	0.363
HSD17B14 (*n*/%)	AA, AG, GG	127 (81.4%), 26 (16.7%), 2 (1.3%)	107 (87.8%), 15 (12.3%), 0 (0%)	0.251
IGF1 (*n*/%)	GG, AG, AA	94 (60.3%), 52 (33.3%), 10 (6.4%)	81 (66.4%), 39 (32.0%), 2 (1.6%)	0.131
IL6 (*n*/%)	CC, GC, GG	20 (12.8%), 71 (45.5%), 65 (41.7%)	26 (21.3%), 61 (50.0%), 35 (28.7%)	0.039
IP6K3 (*n*/%)	TT, TC, CC	0 (0%), 31 (19.9%), 125 (80,1%)	0 (0%), 14 (11.5%), 108 (88.5%)	0.059
ITPR1 (*n*/%)	GG, TG, TT	44 (28.2%), 82 (52.6%), 30 (19.2%)	36 (29.5%), 67 (54.9%), 19 (15.6%)	0.730
**MPRIP** (*n*/%)	GG, AG, AA	102 (65.4%), 52 (33.3%), 2 (1.3%)	95 (77.9%), 20 (16.4%), 7 (5.7%)	**0.001**
MTHFR (*n*/%)	TT, TG, GG	66 (42.3%), 73 (46.8%), 17 (10.9%)	58 (47.5%), 55 (45.1%), 9 (7.4%)	0.504
MTR (*n*/%)	AA, AG, GG	102 (65.4%), 43 (27.6%), 11 (7.1%)	75 (61.5%), 43 (35.2%), 4 (3.3%)	0.194
MTRR (*n*/%)	AA, AG, GG	29 (18.6%), 78 (50.0%), 49 (31.4%)	21 (17.2%), 64 (52.5%), 37 (30.3%)	0.914
NOS3 (*n*/%)	CC, CT, TT	18 (11.5%), 81 (51.9%), 57 (36.5%)	16 (13.1%), 60 (49.2%), 46 (37.7%)	0.876
PPARA (*n*/%)	GG, CG, CC	94 (60.3%), 59 (37.8%), 2 (1.3%)	70 (57.4%), 45 (36.9%), 7 (5.7%)	0.116
PPARG (*n*/%)	CC, CG, GG	130 (83.3%), 24 (15.4%), 2 (1.3%)	88 (72.1%), 32 (26.2%), 2 (1.6%)	0.076
TRHR (*n*/%)	CC, TC, TT	74 (47.4%), 71 (45.5%), 11 (7.1%)	69 (56.6%), 37 (30.3%), 16 (13.1%)	0.021
UCP2 (*n*/%)	AA, AG, GG	34 (21.8%), 73 (46.8%), 49 (31.4%)	24 (19.7%), 48 (39.3%), 50 (41.0%)	0.249
VDR (*n*/%)	CC, TC, TT	59 (37.8%), 70 (44.9%), 27 (17.3%)	50 (41.0%), 56 (45.9%), 16 (13.1%)	0.616

Note: Significant differences between male and female athletes after Bonferroni-correction (p < 0.002) are displayed in bold.

### Genotype-Phenotype Study

Correlations between single SNPs with the CMJ and 10-m sprint performance are displayed in Table 3. For male athletes, a significant correlation was found between AGT (ρ = 0.228, *p* = 0.005) and the Vitamin D receptor rs1544410 polymorphism (VDR; ρ = 0.165, *p* = 0.042) with the CMJ as well as between IP6K3 (ρ = −0.251, *p* = 0.014) and 10-m sprint performance. For female athletes, a significant correlation was found between ACTN3 (ρ = 0.231, *p* = 0.012), AGT (ρ = 0.208, *p* = 0.024), UCP2 (ρ = −0.199, *p* = 0.031), and the CMJ as well as between HSD17B14 (ρ = −0.398, *p* = 0.002), MTRR (ρ = 0.216, *p* = 0.045), UCP2 (ρ = 0.294, *p* = 0.002) and 10-m sprint performance.

**Table 3:** Correlations between single SNPs with the countermovement jump (CMJ) and sprint performance for male and female athletes.

Gene variant (SNP)	CMJ − male		Sprint − male		CMJ − female		Sprint − female	
	*p*	ρ	*p*	ρ	*p*	ρ	*p*	ρ
ACE	0.401	−0.068	0.386	0.074	0.747	0.030	0.189	−0.127
**ACTN3**	0.975	−0.003	0.761	0.026	**0.012**	**0.231**	0.223	−0.118
ADRB2	0.512	0.053	0.130	−0.129	0.140	0.137	0.331	−0.095
**AGT**	**0.005**	**0.228**	0.095	−0.142	**0.024**	**0.208**	0.598	−0.051
IL6	0.101	0.101	0.685	−0.035	0.835	0.019	1.000	0.000
NOS3	0.839	−0.017	0.474	−0.061	0.555	−0.055	0.600	0.051
TRHR	0.114	−0.128	0.499	0.058	0.608	0.048	0.932	−0.008
PPARA	0.290	−0.086	0.349	0.080	0.687	−0.037	0.577	−0.054
COTL1	0.342	0.077	0.103	−0.139	0.763	0.028	0.288	−0.103
CPNE5	0.760	0.025	0.852	0.016	0.347	0.087	0.154	−0.138
DMD	0.439	−0.063	0.627	0.042	0.461	−0.068	0.474	0.070
HIF1A	0.802	0.020	0.580	−0.047	0.844	0.018	0.568	−0.056
**HSD17B14**	0.845	0.016	0.258	0.097	0.574	0.052	**0.045**	**−0.194**
IGF1	0.534	0.051	0.981	−0.002	0.761	−0.028	0.231	−0.116
**IP6K3**	0.281	0.088	**0.042**	**−0.172**	0.360	0.085	0.712	0.036
ITPR1	0.509	0.054	0.189	−0.112	0.703	0.036	0.749	−0.031
MPRIP	0.065	0.150	1.000	0.000	0.104	−0.150	0.819	0.022
MTHFR	0.837	0.017	0.287	−0.091	0.541	−0.057	0.898	−0.012
MTR	0.795	−0.021	0.090	−0.144	0.255	0.106	0.088	−0.165
**MTRR**	0.171	0.111	0.842	−0.017	0.512	−0.061	**0.025**	**0.216**
PPARG	0.766	0.024	0.784	−0.023	0.790	0.025	0.420	0.078
**UCP2**	0.821	0.018	0.429	0.068	**0.031**	**−0.199**	**0.002**	**0.294**
**VDR**	**0.042**	**0.165**	0. 215	−0.106	0.947	0.006	0.871	−0.016

Note: Significant correlations are displayed in bold.

### Polygenic Study

TGSsig ranged from 16.67 to 100.0 a.u. (including three SNPs: AGT, VDR & IP6K3) in male and from 0.00 to 70.0 a.u. (including five SNPs: ACTN3, AGT, HSD17B14, MTRR & UCP2) in female athletes. TGSsig showed a significant correlation with the CMJ and sprint performance in males (CMJ *r* = 0.328, *p* < 0.001; 10-m sprint *r* = −0.241, *p* = 0.004) as well as in females (CMJ *r* = 0.320, *p* < 0.001; 10-m sprint *r* = −0.320, *p* < 0.001) indicating a moderate correlation (Cohen, 1988).

**Figure 1:**
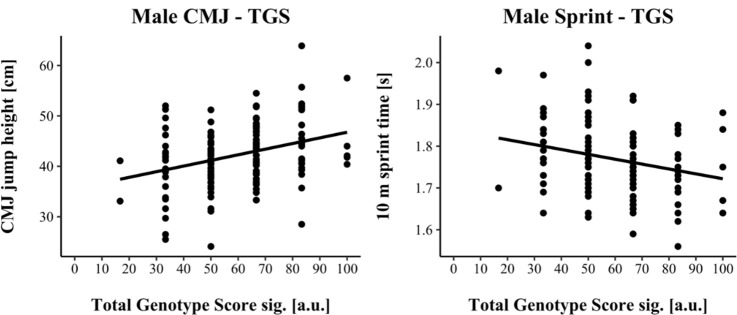
Scatterplot between TGSsig with the countermovement jump (CMJ) and sprint performance for male athletes.

**Figure 2:**
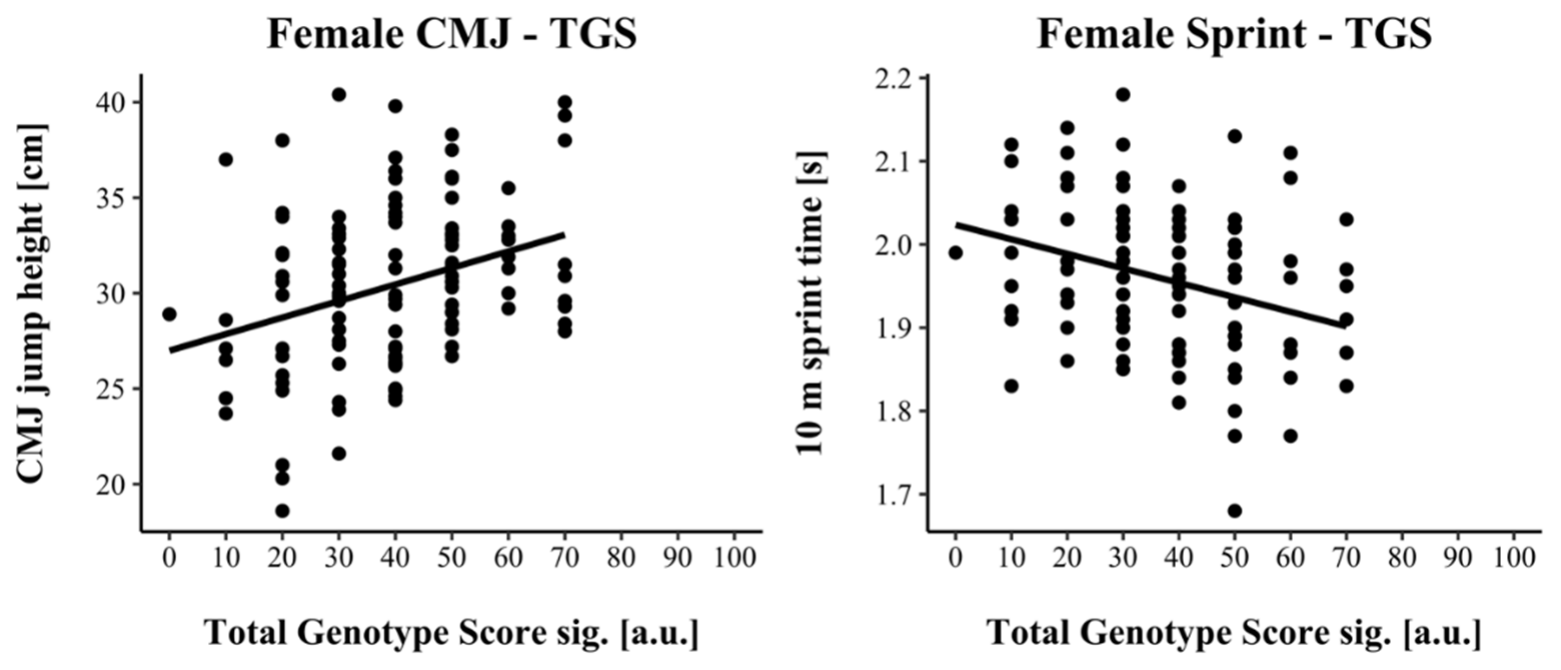
Scatterplot between TGSsig with the countermovement jump (CMJ) and sprint performance for female athletes.

For linear regression, male results are shown in Figure 1, while female results are shown in Figure 2. Assumptions for performing linear regression were checked by visual inspection (linearity, normality, and homoscedasticity). Autocorrelation of residuals was verified using the Durbin-Watson statistic (male: CMJ = 1.50, sprint = 1.68; female: CMJ = 1.25, sprint = 1.18). For male athletes, the overall model indicated a moderate goodness-of-fit for the CMJ (*R^2^* = 0.11, adjusted *R^2^* = 0.10) and a small to moderate goodness-of-fit for the 10-m sprint prediction (*R^2^* = 0.06, adjusted *R^2^* = 0.05) according to Cohen (1988). TGSsig could significantly predict the CMJ (*F*(1, 151) = 18.17, *p* < 0.001) and 10-m sprint performance (*F*(1, 137) = 8.47, *p* = 0.004). For female athletes, the overall model indicated a moderate goodness-of-fit for the CMJ (*R^2^* = 0.10, adjusted *R^2^* = 0.09) as well as for the 10-m sprint prediction (*R^2^* = 0.10, adjusted *R^2^* = 0.09) according to Cohen (1988). TGSsig could significantly predict the CMJ (*F*(1, 116) = 13.27, *p* < 0.001), and 10-m sprint performance (*F*(1, 106) = 12.10, *p* < 0.001).

## Discussion

The aim of the study was to answer the question to what extent the genotype can predict phenotypical power performance in elite athletes. Power is a key variable for peak performance in both team and individual sports and can differentiate between elite and non-elite athletes (Tsoukos et al., 2019). Therefore, it seems useful to monitor power performance on a regular basis as well as to improve power performance. Previous studies showed a relationship between SNPs such as ACTN3 or ACE and power performance, and were able to predict power performance based on polygenic scores (Petr et al., 2022). Our findings confirm this genotype-phenotype relationship. Polygenic score regression used in our study explained around 10% of the variance in power performance independently of gender. This is consistent with our expectations and in line with findings from the study by Petr et al. (2022), demonstrating that polygenic score regression explained even 26% of variance in power performance. Compared to their work, slightly less variance was explained in our study. These differences may partly be explained by the dependent variable chosen in the study. While Petr et al. (2022) used sergeant jump and isokinetic strength measures as dependent power variables, we focused on CMJ and sprint measures. When comparing our findings with other domains such as cognition, Davies et al. (2018) predicted up to 4.3% of variance in general cognitive function using polygenic scores, whereas we could explain variance to a greater extent. Our findings strengthen the idea of power being partly heritable which in our study amounts to 10% of explained variance in performance by genotype. Obviously, other aspects such as training modalities as well as other individual features (Ullén et al., 2016) determine power performance.

When investigating the relationship between single SNPs and power performance, our results showed a significant correlation of AGT and VDR with the CMJ as well as between IP6K3 and sprint performance in male athletes. For female athletes, a relationship was found between ACTN3, AGT, UCP2 and the CMJ as well as between HSD17B14, MTRR, UCP2 and sprint performance. These findings are in line with previous studies reporting associations of SNPs with power performance and power athlete status for AGT (Gómez-Gallego et al., 2009b), VDR bsml (Bollen et al., 2023; Bozsodi et al., 2016), IP6K3 (Maciejewska-Skrendo et al., 2019), ACTN3 (Petr et al., 2022; Yang et al., 2003), and HSD17B14 (Pickering et al., 2019). Sessa et al. (2011) found the C allele of the UCP2 gene polymorphism (rs660339) to be over-represented among Italian power athletes. Further, Terruzzi et al. (2011) found a higher frequency of the G allele in the A66G polymorphism of MTRR (rs1801394) in athletes compared to controls indicating that athletes had a genetic predisposition for muscle growth. In contrast, we found significant correlations between the T allele in UCP2 and the A allele in MTRR with faster sprint times. Yet, this is the first study combining UCP2 and MTRR genotypes with power performance phenotypes in elite athletes indicating that the T allele in UCP2 and the A allele in MTRR might also be related to power performance in an elite athletes’ population.

No significant correlations were found for the following SNPs neither in male nor in female athletes: ACE, Adrenoceptor beta 2 - ADRB2 Gly16Arg, Coactosin-like protein 1 - COTL1 rs7458, Copine V - CPNE5 rs3213537, DMD, Hypoxia inducible factor 1 subunit alpha - HIF1A, Insulin-like growth factor 1 - IGF1 Pro582Ser, Interleukin 6 - IL6 –174 G/C, Inositol 1,4,5-triphosphate receptor type 1 - ITPR1 rs1038639, MPRIP rs6502557, Methylenetetrahydrofolate reductase - MTHFR A1298C, 5-methyltetrahydrofolate-homocysteine methyltransferase - MTR A2756G, Nitric oxide synthase 3 - NOS3 –786 T/C polymorphism, Peroxisome proliferator-activated receptor alpha - PPARA rs4253778, Peroxisome proliferator-activated receptor gamma - PPARG Pro12Ala, and Thyrotropin-releasing hormone receptor - TRHR rs7832552. This is only partly consistent with previous studies in which correlations between these SNPs and power performance have been reported (Table 1 for specific references). However, our findings add to the literature given that there are inconsistencies in the relationship between SNPs and power performance variables for the mentioned SNPs. Although ACE is a well-studied polymorphism with studies indicating a relationship with endurance and power performance (Ahmetov et al., 2022), Yang et al. (2023), for example, did not find significant correlations between ACE as well as ACTN3 polymorphisms and muscle power in Chinese elite and sub-elite athletes. In addition, some relations are based on power athlete status in which the genotype was compared between elite and sub-elite athletes without relating SNPs to objective power performance variables. As described by Hagberg et al. (2011), small sample sizes may also be limiting which is, however, an eminent factor in the elite athletes’ population.

Furthermore, some gender-specific aspects emerged in our analyses. While there was a significant relationship between AGT and CMJ performance in males as well as in females, some relationships were only evident in male but not in female athletes, and vice versa. For example, ACTN3 was only related to CMJ performance in females but not in males. This indicates that the relationship between specific SNPs and phenotypical power performance may differ between genders. This observation is in line with previous studies (Landen et al., 2019). For example, Min et al. (2009) found associations between ACE and race distance in male but not in female athletes. In accordance with this, Willems et al. (2017) found stronger associations between a polygenic score and grip strength in males compared to females. However, when looking at the results of our regression analysis, the explained variance did not differ substantially between genders, since we were able to explain 10% of variance in the CMJ and sprint performance in both genders. Thus, differences between sexes are not completely clear and should be addressed in future investigations. Concerning genotype frequencies, a difference between genders was shown in DMD and MPRIP. Although we only found a small effect for MPRIP, our analyses show a large effect for DMD. Since DMD is located on the X-chromosome (sex chromosome; Monaco et al., 1986), no heterozygous genotype exists in males. While females carry two X-chromosomes and males one X- and one Y-chromosome, no heterozygous genotypes can be found as the genetic information of the second X-chromosome is missing.

In summary our findings further support the idea that performance is partly heritable. However, our results suggest that the genotype only partially predicts power performance. Factors such as training modalities (i.e., the way athletes train) may play a crucial role in power performance. Given the importance of power for elite performance, the primary goal in elite sports is to maximize power performance. This is also one of the objectives of the in:prove project, in which these findings are to be used in future to develop individualized training prescriptions. Such an approach is in line with previous research that has shown the benefits of tailored training prescriptions considering the athletes’ genotype (Jones et al., 2016). Further longitudinal studies are required to investigate the role of the genotype in individual training adaptations as well as the relationship with the existing field approaches (e.g., force-velocity profiling; Morin and Samozino, 2016).

## Conclusions

Significant correlations were found between ACTN3, AGT, UCP2, VDR and the CMJ as well as between HSD17B14, IP6K3, MTRR, UCP2 and 10-m sprint performance. The athletes’ genotype could explain 10% of variance in power performance. This strengthens the idea of power being partly heritable, however, results indicate that the genotype only partially, by 10%, determines power performance. The role of the genotype in the individual performance development should be investigated in future studies.

## References

[ref1] Ahmetov, I. I., and Fedotovskaya, O. N. (2015). Current progress in sports genomics. Advances in Clinical Chemistry, 70, 247–314. 10.1016/bs.acc.2015.03.003 26231489

[ref2] Ahmetov, I. I., Hall, E. C. R., Semenova, E. A., Pranckevičienė, E., and Ginevičienė, V. (2022). Advances in sports genomics. Advances in Clinical Chemistry, 107, 215–263. 10.1016/bs.acc.2021.07.004 35337603

[ref3] Appel, M., Zentgraf, K., Krüger, K., and Alack, K. (2021). Effects of genetic variation on endurance performance, muscle strength, and injury susceptibility in sports: a systematic review. *Frontiers in Physiology*, 12, 694411. 10.3389/fphys.2021.694411 34366884 PMC8334364

[ref4] Ben-Zaken, S., Meckel, Y., Nemet, D., and Eliakim, A. (2013). Can IGF-I polymorphism affect power and endurance athletic performance? Growth Hormone & IGF Research, 23(5), 175–178. 10.1016/j.ghir.2013.06.005 23850449

[ref5] Bollen, S. E., Bass, J. J., Wilkinson, D. J., Hewison, M., and Atherton, P. J. (2023). The impact of genetic variation within the vitamin D pathway upon skeletal muscle function: a systematic review. Journal of Steroid Biochemistry and Molecular Biology, 229, 106266. 10.1016/j.jsbmb.2023.106266 36822332

[ref6] Bozsodi, A., Boja, S., Szilagyi, A., Somhegyi, A., Varga, P. P., and Lazary, A. (2016). Muscle strength is associated with vitamin D receptor gene variants. Journal of Orthopaedic Research, 34(11), 2031–2037. 10.1002/jor.23220 26932507

[ref7] Cohen, J. (1988). *Statistical Power Analysis for the Behavioral Sciences* (2^nd^ ed.). Routledge. 10.4324/9780203771587

[ref8] Cronin, J., and Sleivert, G. (2005). Challenges in understanding the influence of maximal power training on improving athletic performance. Sports Medicine, 35(3), 213–234. 10.2165/00007256-200535030-00003 15730337

[ref9] Davies, G., Lam, M., Harris, S. E., Trampush, J. W., Luciano, M., Hill, W. D., Hagenaars, S. P., Ritchie, S. J., Marioni, R. E., Fawns-Ritchie, C., Liewald, D. C. M., Okely, J. A., Ahola-Olli, A. V., Barnes, C. L. K., Bertram, L., Bis, J. C., Burdick, K. E., Christoforou, A., DeRosse, P., Djurovic, S., … Deary, I. J. (2018). Study of 300,486 individuals identifies 148 independent genetic loci influencing general cognitive function. *Nature Communications*, 9(1), 2098. 10.1038/s41467-018-04362-x PMC5974083 29844566

[ref10] Del Coso, J., Hiam, D., Houweling, P., Pérez, L. M., Eynon, N., and Lucía, A. (2019). More than a 'speed gene': ACTN3 R577X genotype, trainability, muscle damage, and the risk for injuries. European Journal of Applied Physiology, 119(1), 49–60. 10.1007/s00421-018-4010-0 30327870

[ref11] Douda, H. T., Toubekis, A. G., Avloniti, A. A., and Tokmakidis, S. P. (2008). Physiological and anthropometric determinants of rhythmic gymnastics performance. International Journal of Sports Physiology and Performance, 3(1), 41–54. 10.1123/ijspp.3.1.41 19193953

[ref12] Drozdovska, S. B., Dosenko, V. E., Ahmetov, I. I., and Ilyin, V. N. (2013). The association of gene polymorphisms with athlete status in Ukrainians. Biology of Sport, 30(3), 163–167. 10.5604/20831862.1059168 24744483 PMC3944573

[ref13] El Ouali, E. M., Barthelemy, B., Del Coso, J., Hackney, A. C., Laher, I., Govindasamy, K., Mesfioui, A., Granacher, U., and Zouhal, H. (2024). A systematic review and meta-analysis of the association between ACTN3 R577X genotypes and performance in endurance versus power athletes and non-athletes. *Sports Medicine - Open*, 10(1), 37. 10.1186/s40798-024-00711-x 38609671 PMC11014841

[ref14] Ericsson, K. A., Krampe, R. T., and Tesch-Römer, C. (1993). The role of deliberate practice in the acquisition of expert performance. Psychological Review, 100(3), 363–406. 10.1037/0033-295X.100.3.363

[ref15] Eynon, N., Alves, A. J., Meckel, Y., Yamin, C., Ayalon, M., Sagiv, M., and Sagiv, M. (2010). Is the interaction between HIF1A P582S and ACTN3 R577X determinant for power/sprint performance? Metabolism: Clinical and Experimental, 59(6), 861–865. 10.1016/j.metabol.2009.10.003 20005538

[ref16] Gómez-Gallego, F., Ruiz, J. R., Buxens, A., Artieda, M., Arteta, D., Santiago, C., Rodríguez-Romo, G., Lao, J. I., and Lucia, A. (2009a). The-786 T/C polymorphism of the NOS3 gene is associated with elite performance in power sports. European Journal of Applied Physiology, 107(5), 565–569. 10.1007/s00421-009-1166-7 19701646

[ref17] Gómez-Gallego, F., Santiago, C., González-Freire, M., Yvert, T., Muniesa, C. A., Serratosa, L., Altmäe, S., Ruiz, J. R., and Lucia, A. (2009b). The C allele of the AGT Met235Thr polymorphism is associated with power sports performance. Applied Physiology, Nutrition, and Metabolism, 34(6), 1108–1111. 10.1139/H09-108 20029521

[ref18] Gonçalves, C. A., Lopes, T. J. D., Nunes, C., Marinho, D. A., and Neiva, H. P. (2021). Neuromuscular jumping performance and upper-body horizontal power of volleyball players. Journal of Strength and Conditioning Research, 35(8), 2236–2241. 10.1519/JSC.0000000000003139 30946267

[ref19] Guest, N. S., Horne, J., Vanderhout, S. M., and El-Sohemy, A. (2019). Sport nutrigenomics: personalized nutrition for athletic performance. *Frontiers in Nutrition*, 6, 8. 10.3389/fnut.2019.00008 30838211 PMC6389634

[ref20] Guilherme, J. P. L. F., Semenova, E. A., Zempo, H., Martins, G. L., Lancha Junior, A. H., Miyamoto-Mikami, E., Kumagai, H., Tobina, T., Shiose, K., Kakigi, R., Tsuzuki, T., Ichinoseki-Sekine, N., Kobayashi, H., Naito, H., Borisov, O. V., Kostryukova, E. S., Kulemin, N. A., Larin, A. K., Generozov, E. V., Fuku, N., … Ahmetov, I. I. (2021). Are genome-wide association study identified single-nucleotide polymorphisms associated with sprint athletic status? A replication study with 3 different cohorts. International Journal of Sports Physiology and Performance, 16(4), 489–495. 10.1123/ijspp.2019-1032 33059329

[ref21] Hagberg, J. M., Rankinen, T., Loos, R. J., Pérusse, L., Roth, S. M., Wolfarth, B., and Bouchard, C. (2011). Advances in exercise, fitness, and performance genomics in 2010. Medicine and Science in Sports and Exercise, 43(5), 743–752. 10.1249/MSS.0b013e3182155d21 21499051 PMC3951763

[ref22] Jones, N., Kiely, J., Suraci, B., Collins, D. J., de Lorenzo, D., Pickering, C., and Grimaldi, K. A. (2016). A genetic-based algorithm for personalized resistance training. Biology of Sport, 33(2), 117–126. 10.5604/20831862.1198210 27274104 PMC4885623

[ref23] Landen, S., Voisin, S., Craig, J. M., McGee, S. L., Lamon, S., and Eynon, N. (2019). Genetic and epigenetic sex-specific adaptations to endurance exercise. Epigenetics, 14(6), 523–535. 10.1080/15592294.2019.1603961 30957644 PMC6557612

[ref24] Ma, F., Yang, Y., Li, X., Zhou, F., Gao, C., Li, M., and Gao, L. (2013). The association of sport performance with ACE and ACTN3 genetic polymorphisms: a systematic review and meta-analysis. *PloS One*, 8(1), e54685. 10.1371/journal.pone.0054685 23358679 PMC3554644

[ref25] Maciejewska-Skrendo, A., Cięszczyk, P., Chycki, J., Sawczuk, M., and Smółka, W. (2019). Genetic markers associated with power athlete status. Journal of Human Kinetics, 68, 17–36. 10.2478/hukin-2019-0053 31531130 PMC6724599

[ref26] Maciejewska-Skrendo, A., Buryta, M., Czarny, W., Król, P., Spieszny, M., Stastny, P., Petr, M., Safranow, K., & Sawczuk, M. (2019). The Polymorphisms of the Peroxisome-Proliferator Activated Receptors’ Alfa Gene Modify the Aerobic Training Induced Changes of Cholesterol and Glucose. Journal of Clinical Medicine, 8(10), 1043. 10.3390/jcm8071043 31319591 PMC6679124

[ref27] Maciejewska-Skrendo, A., Mieszkowski, J., Kochanowicz, A., Niespodziński, B., Cieszczyk, P., Leźnicka, K., Leońska-Duniec, A., Kolbowicz, M., Kaczmarczyk, M., Piskorska, E., Stankiewicz, B., Stępniak, R., Mostowik, A., Zawartka, M., Rzeszutko-Bełzowska, A., Massidda, M., Caló, C. M., Kemerytė-Riaubienė, E., and Sawczuk, M. (2021). Does the *PPARA* intron 7 gene variant (rs4253778) influence performance in power/strength-oriented athletes? A case-control replication study in three cohorts of European gymnasts. Journal of Human Kinetics, 79, 77–85. 10.2478/hukin-2020-0060 34400988 PMC8336554

[ref28] Markovic, G., Dizdar, D., Jukic, I., and Cardinale, M. (2004). Reliability and factorial validity of squat and countermovement jump tests. Journal of Strength and Conditioning Research, 18(3), 551–555. 10.1519/1533-4287(2004)18<551:RAFVOS>2.0.CO;2 15320660

[ref29] McAuley, A. B. T., Hughes, D. C., Tsaprouni, L. G., Varley, I., Suraci, B., Bradley, B., Baker, J., Herbert, A. J., and Kelly, A. L. (2024). Genetic associations with acceleration, change of direction, jump height, and speed in English academy football players. Journal of Strength and Conditioning Research, 38(2), 350–359. 10.1519/JSC.0000000000004634 38258831

[ref30] Mero, A., Komi, P. V., and Gregor, R. J. (1992). Biomechanics of sprint running. A review. Sports Medicine, 13(6), 376–392. 10.2165/00007256-199213060-00002 1615256

[ref31] Min, S. K., Takahashi, K., Ishigami, H., Hiranuma, K., Mizuno, M., Ishii, T., Kim, C. S., and Nakazato, K. (2009). Is there a gender difference between ACE gene and race distance? Applied Physiology, Nutrition, and Metabolism, 34(5), 926–932. 10.1139/H09-097 19935855

[ref32] Miyamoto-Mikami, E., Murakami, H., Tsuchie, H., Takahashi, H., Ohiwa, N., Miyachi, M., Kawahara, T., and Fuku, N. (2017). Lack of association between genotype score and sprint/power performance in the Japanese population. Journal of Science and Medicine in Sport, 20(1), 98–103. 10.1016/j.jsams.2016.06.005 27380726

[ref33] Monaco, A. P., Neve, R. L., Colletti-Feener, C., Bertelson, C. J., Kurnit, D. M., and Kunkel, L. M. (1986). Isolation of candidate cDNAs for portions of the Duchenne muscular dystrophy gene. Nature, 323(6089), 646–650. 10.1038/323646a0 3773991

[ref34] Moreland, E., Borisov, O. V., Semenova, E. A., Larin, A. K., Andryushchenko, O. N., Andryushchenko, L. B., Generozov, E. V., Williams, A. G., and Ahmetov, I. I. (2022). Polygenic profile of elite strength athletes. Journal of Strength and Conditioning Research, 36(9), 2509–2514. 10.1519/JSC.0000000000003901 33278272

[ref35] Morin, J. B., and Samozino, P. (2016). Interpreting power-force-velocity profiles for individualized and specific training. International Journal of Sports Physiology and Performance, 11(2), 267–272. 10.1123/ijspp.2015-0638 26694658

[ref36] Petr, M., Thiel, D., Kateřina, K., Brož, P., Malý, T., Zahálka, F., Vostatková, P., Wilk, M., Chycki, J., and Stastny, P. (2022). Speed and power-related gene polymorphisms associated with playing position in elite soccer players. Biology of Sport, 39(2), 355–366. 10.5114/biolsport.2022.105333 35309536 PMC8919892

[ref37] Pickering, C., Suraci, B., Semenova, E. A., Boulygina, E. A., Kostryukova, E. S., Kulemin, N. A., Borisov, O. V., Khabibova, S. A., Larin, A. K., Pavlenko, A. V., Lyubaeva, E. V., Popov, D. V., Lysenko, E. A., Vepkhvadze, T. F., Lednev, E. M., Leońska-Duniec, A., Pająk, B., Chycki, J., Moska, W., Lulińska-Kuklik, E., … Ahmetov, I. I. (2019). A genome-wide association study of sprint performance in elite youth football players. Journal of Strength and Conditioning Research, 33(9), 2344–2351. 10.1519/JSC.0000000000003259 31343553

[ref38] Puthucheary, Z., Skipworth, J. R., Rawal, J., Loosemore, M., Van Someren, K., and Montgomery, H. E. (2011). The ACE gene and human performance: 12 years on. Sports Medicine, 41(6), 433–448. 10.2165/11588720-000000000-00000 21615186

[ref39] Ransdell, L. B., and Murray, T. (2011). A physical profile of elite female ice hockey players from the USA. Journal of Strength and Conditioning Research, 25(9), 2358–2363. 10.1519/JSC.0b013e31822a5440 21804420

[ref40] Ruiz, J. R., Arteta, D., Buxens, A., Artieda, M., Gómez-Gallego, F., Santiago, C., Yvert, T., Morán, M., and Lucia, A. (2010a). Can we identify a power-oriented polygenic profile? Journal of Applied Physiology, 108(3), 561–566. 10.1152/japplphysiol.01242.2009 20044471

[ref41] Ruiz, J. R., Buxens, A., Artieda, M., Arteta, D., Santiago, C., Rodríguez-Romo, G., Lao, J. I., Gómez-Gallego, F., and Lucia, A. (2010b). The-174 G/C polymorphism of the IL6 gene is associated with elite power performance. Journal of Science and Medicine in Sport, 13(5), 549–553. 10.1016/j.jsams.2009.09.005 19853505

[ref42] Sawczuk, M., Maciejewska-Karlowska, A., Cieszczyk, P., Skotarczak, B., and Ficek, K. (2013). Association of the ADRB2 Gly16Arg and Glu27Gln polymorphisms with athlete status. Journal of Sports Sciences, 31(14), 1535–1544. 10.1080/02640414.2013.786184 23631811

[ref43] Sessa, F., Chetta, M., Petito, A., Franzetti, M., Bafunno, V., Pisanelli, D., Sarno, M., Iuso, S., and Margaglione, M. (2011). Gene polymorphisms and sport attitude in Italian athletes. Genetic Testing and Molecular Biomarkers, 15(4), 285–290. 10.1089/gtmb.2010.0179 21254885

[ref44] Terruzzi, I., Senesi, P., Montesano, A., La Torre, A., Alberti, G., Benedini, S., Caumo, A., Fermo, I., and Luzi, L. (2011). Genetic polymorphisms of the enzymes involved in DNA methylation and synthesis in elite athletes. Physiological Genomics, 43(16), 965–973. 10.1152/physiolgenomics.00040.2010 21673074

[ref45] Tsoukos, A., Drikos, S., Brown, L. E., Sotiropoulos, K., Veligekas, P., and Bogdanis, G. C. (2019). Upper and lower body power are strong predictors for selection of male junior national volleyball team players. Journal of Strength and Conditioning Research, 33(10), 2760–2767. 10.1519/JSC.0000000000002472 29385001

[ref46] Ullén, F., Hambrick, D. Z., and Mosing, M. A. (2016). Rethinking expertise: a multifactorial gene-environment interaction model of expert performance. Psychological Bulletin, 142(4), 427–446. 10.1037/bul0000033 26689084

[ref47] Varillas Delgado, D., Del Coso, J., Gutiérrez-Hellín, J., Aguilar-Navarro, M., Muñoz, A., Maestro, A., and Morencos, E. (2022). Genetics and sports performance: the present and future in the identification of talent for sports based on DNA testing. European Journal of Applied Physiology, 122(8), 1811–1830. 10.1007/s00421-022-04945-z 35428907 PMC9012664

[ref48] Vigh-Larsen, J. F., Beck, J. H., Daasbjerg, A., Knudsen, C. B., Kvorning, T., Overgaard, K., Andersen, T. B., and Mohr, M. (2019). Fitness characteristics of elite and subelite male ice hockey players: a cross-sectional study. Journal of Strength and Conditioning Research, 33(9), 2352–2360. 10.1519/JSC.0000000000003285 31343551

[ref49] Williams, A. G., and Folland, J. P. (2008). Similarity of polygenic profiles limits the potential for elite human physical performance. Journal of Physiology, 586(1), 113–121. 10.1113/jphysiol.2007.141887 17901117 PMC2375556

[ref50] Willems, S. M., Wright, D. J., Day, F. R., Trajanoska, K., Joshi, P. K., Morris, J. A., Matteini, A. M., Garton, F. C., Grarup, N., Oskolkov, N., Thalamuthu, A., Mangino, M., Liu, J., Demirkan, A., Lek, M., Xu, L., Wang, G., Oldmeadow, C., Gaulton, K. J., Lotta, L. A., … Scott, R. A. (2017). Large-scale GWAS identifies multiple loci for hand grip strength providing biological insights into muscular fitness. *Nature Communications*, 8, 16015. 10.1038/ncomms16015 PMC5510175 29313844

[ref51] Yang, N., MacArthur, D. G., Gulbin, J. P., Hahn, A. G., Beggs, A. H., Easteal, S., and North, K. (2003). ACTN3 genotype is associated with human elite athletic performance. American Journal of Human Genetics, 73(3), 627–631. 10.1086/377590 12879365 PMC1180686

[ref52] Yang, S., Lin, W., Jia, M., and Chen, H. (2023). Association between ACE and ACTN3 genes polymorphisms and athletic performance in elite and sub-elite Chinese youth male football players. *PeerJ*, 11, e14893. 10.7717/peerj.14893 36992938 PMC10042156

[ref53] Zarębska, A., Ahmetov, I. I., Sawczyn, S., Weiner, A. S., Kaczmarczyk, M., Ficek, K., Maciejewska-Karlowska, A., Sawczuk, M., Leonska-Duniec, A., Klocek, T., Voronina, E. N., Boyarskikh, U. A., Filipenko, M. L., and Cieszczyk, P. (2014). Association of the MTHFR 1298A>C (rs1801131) polymorphism with speed and strength sports in Russian and Polish athletes. Journal of Sports Sciences, 32(4), 375–382. 10.1080/02640414.2013.825731 24015812

[ref54] Zarębska, A., Sawczyn, S., Kaczmarczyk, M., Ficek, K., Maciejewska-Karłowska, A., Sawczuk, M., Leońska-Duniec, A., Eider, J., Grenda, A., and Cięszczyk, P. (2013). Association of rs699 (M235T) polymorphism in the AGT gene with power but not endurance athlete status. Journal of Strength and Conditioning Research, 27(10), 2898–2903. 10.1519/JSC.0b013e31828155b5 23287839

[ref55] Zentgraf, K., and Raab, M. (2023). Excellence and expert performance in sports: what do we know and where are we going? International Journal of Sport and Exercise Psychology, 21(5), 766–786. 10.1080/1612197X.2023.2229362

